# Knowledge of dental academics about the COVID-19 pandemic: a multi-country online survey

**DOI:** 10.1186/s12909-020-02308-w

**Published:** 2020-11-02

**Authors:** Nour Ammar, Nourhan M. Aly, Morenike O. Folayan, Simin Z. Mohebbi, Sameh Attia, Hans-Peter Howaldt, Sebastian Boettger, Yousef Khader, Diah A. Maharani, Anton Rahardjo, Imran Khan, Marwa Madi, Anas Shamala, Ola B. Al-Batayneh, Maher Rashwan, Verica Pavlic, Smiljka Cicmil, Gabriella Galluccio, Antonella Polimeni, Davide Mancino, Arheiam Arheiam, Mai A. Dama, Myat Nyan, Prathip Phantumvanit, Jin-Bom Kim, Youn-Hee Choi, Jorge L. Castillo, Easter Joury, Maha M. Abdelsalam, Mohammad M. Alkeshan, Iyad Hussein, Ana P. Vukovic, Alfredo Iandolo, Arthur M. Kemoli, Maha El Tantawi

**Affiliations:** 1 Department of Pediatric Dentistry and Dental Public Health, Faculty of Dentistry, Alexandria University, Alexandria, Egypt; 2 Department of Child Dental Health, Obafemi Awolowo University, Ile-Ife, Nigeria; 3 Research Center for Caries Prevention, Dentistry Research Institute, Tehran University of Medical Sciences, Tehran, Iran; 4 Community Oral Health Department, School of Dentistry, Tehran University of Medical Sciences, Tehran, Iran; 5 Department of Cranio-Maxillofacial Surgery, Justus-Liebig University Giessen, Giessen, Germany; 6 Department of Public Health, Jordan University of Science and Technology, Irbid, Jordan; 7 Department of Preventive and Public Health Dentistry, Faculty of Dentistry, Universitas Indonesia, Depok, Indonesia; 8 Department of Oral & Maxillofacial Surgery, Faculty of Dentistry, Jamia Millia Islamia, New Delhi, India; 9 Department of Preventive Dental Sciences, College of Dentistry, Imam Abdulrahman Bin Faisal University, Dammam, Saudi Arabia; 10 Department of Preventive and Biomedical Science, College of Dentistry, University of Science & Technology, Sanaa, Yemen; 11 Department of Preventive Dentistry, Faculty of Dentistry, Jordan University of Science and Technology, Irbid, Jordan; 12 Center for Oral Bioengineering, Barts and the London, School of Medicine and Dentistry, Queen Mary University of London, London, UK; 13 Department of Conservative Dentistry, Faculty of Dentistry, Alexandria University, Alexandria, Egypt; 14 Department of Periodontology and Oral Medicine, Medical Faculty University of Banja Luka, Banja Luka, Bosnia and Herzegovina; 15 Department of Oral Rehabilitation, Faculty of Medicine Foca, University of East Sarajevo, East Sarajevo, Bosnia and Herzegovina; 16 Department of Oral and Maxillo Facial Sciences, Faculty of Medicine and Dentistry, Sapienza University of Rome, Rome, Italy; 17 Department of Endodontics and Conservative Dentistry, Faculty of Dental Medicine, University of Strasbourg, 67000 Strasbourg, France; 18 Department of Biomaterials and Bioengineering, INSERM UMR_S 1121, Strasbourg University, 67000 Strasbourg, France; 19 Department of Community and Preventive Dentistry, Faculty of Dentistry, University of Benghazi, Benghazi, Libya; 20 Orthodontics and Pediatric Dentistry Department, Faculty of Dentistry, Arab American University, Jenin, Palestine; 21 Department of Prosthodontics, University of Dental Medicine, Mandalay, Myanmar; 22 Faculty of Dentistry, Thammasat University, Bangkok, Thailand; 23 Department of Preventive and Community Dentistry, School of Dentistry, Pusan National University, Yangsan, Republic of Korea; 24 Department of Preventive Dentistry, School of Dentistry, Kyungpook National University, Daegu, Republic of Korea; 25 Department of Dentistry for Children and Adolescents, Universidad Peruana Cayetano Heredia, Lima, Peru; 26 Centre for Dental Public Health and Primary Care, Institute of Dentistry, Barts and The London School of Medicine and Dentistry, Queen Mary University of London, London, UK; 27 Department of Biomedical Dental Sciences, College of Dentistry, Imam Abdulrahman Bin Faisal University, Dammam, Saudi Arabia; 28 Department of Pediatric Dentistry, Seoul National University Dental Hospital, Seoul, South Korea; 29 Department of Pediatric Dentistry, Mohammed Bin Rashid University of Medicine and Health Sciences, Dubai, United Arab Emirates; 30 Department of Pediatric and Preventive Dentistry, School of Dental Medicine, University of Belgrade, Belgrade, Serbia; 31 Department of Endodontics, University of Salerno, Fisciano, Italy; 32 Department of Paediatric Dentistry & Orthodontics, School of Dental Sciences, University of Nairobi, Nairobi, Kenya

**Keywords:** Dental faculty, COVID-19, Epidemics, Surveys and questionnaires, Multilevel analysis

## Abstract

**Background:**

COVID-19 is a global pandemic affecting all aspects of life in all countries. We assessed COVID-19 knowledge and associated factors among dental academics in 26 countries.

**Methods:**

We invited dental academics to participate in a cross-sectional, multi-country, online survey from March to April 2020. The survey collected data on knowledge of COVID-19 regarding the mode of transmission, symptoms, diagnosis, treatment, protection, and dental treatment precautions as well as participants’ background variables. Multilevel linear models were used to assess the association between dental academics’ knowledge of COVID-19 and individual level (personal and professional) and country-level (number of COVID-19 cases/ million population) factors accounting for random variation among countries.

**Results:**

Two thousand forty-five academics participated in the survey (response rate 14.3%, with 54.7% female and 67% younger than 46 years of age). The mean (SD) knowledge percent score was 73.2 (11.2) %, and the score of knowledge of symptoms was significantly lower than the score of knowledge of diagnostic methods (53.1 and 85.4%, *P* <  0.0001). Knowledge score was significantly higher among those living with a partner/spouse than among those living alone (regression coefficient (B) = 0.48); higher among those with PhD degrees than among those with Bachelor of Dental Science degrees (B = 0.48); higher among those seeing 21 to 30 patients daily than among those seeing no patients (B = 0.65); and higher among those from countries with a higher number of COVID-19 cases/million population (B = 0.0007).

**Conclusions:**

Dental academics had poorer knowledge of COVID-19 symptoms than of COVID-19 diagnostic methods. Living arrangements, academic degrees, patient load, and magnitude of the epidemic in the country were associated with COVD-19 knowledge among dental academics. Training of dental academics on COVID-19 can be designed using these findings to recruit those with the greatest need.

## Background

The 2019 novel coronavirus disease (COVID-19) is a viral respiratory infectious disease caused by the severe acute respiratory syndrome coronavirus 2 (SARS-CoV-2) [1] that is causing a pandemic. The total number of COVID-19 cases reported until May 22nd, 2020, was 5,279,643, with 338,666 deaths and 213 countries affected [2]. Like most respiratory infections, SARS-CoV-2 is transmitted through respiratory droplets, direct contact, and possibly through aerosol-generating procedures such as many dental procedures [3]. There have also been calls to assess the risk of transmission through saliva [4], and whether asymptomatic and pre-symptomatic patients can transmit the infection [3].

Symptoms of COVID-19 infection vary from mild-to-severe fever, dry cough, shortness of breath, fatigue, and atypical symptoms, such as muscle pain, confusion, headache, sore throat, diarrhea, and vomiting. Moderate-to-severe symptoms, such as severe acute respiratory distress, may progress to respiratory failure and death [5]. A significant number of patients with COVID-19 also present with loss of smell and taste [6], which may prompt them to consult a dentist for care.

COVID-19 is of interest to dentists because of the risk of infection in their practices. Dental practitioners can inhale aerosol/ droplets from infected asymptomatic patients or through direct contact with mucous membranes, oral fluids, contaminated instruments, and surfaces. Effective infection-control practices, such as good hand hygiene, disinfection of all surfaces in the clinic, use of personal protective equipment (including masks, gloves, gowns, and goggles or face shields), and specifically, the use of N-95 masks for routine dental practice are recommended precautions [3, 7].

COVID-19 has affected dentists and dental academics not only because of personal fear of contracting the disease or passing it to loved ones and others, but also because of worries about their ability to carry out their academic and research responsibilities in addition to stresses due to restricted mobility [8]. In addition, the temporary closure of dental schools and suspension of dental care services has added to their worries about their ability to provide optimal training for their students. Fear of contracting SARS-CoV-2 infection is a primary concern for both dental academics and their students. Therefore, dental academics need to be conversant with details on transmission, symptoms, treatment, diagnosis, and dental treatment precautionary measures of the disease.

Recent studies have assessed dentists’ knowledge of COVID-19 in Jordan [9], Pakistan [10], and other countries [11]. Dentists’ fears [12] and their challenges with offering dental treatment during the pandemic have also been assessed [7]. However, none of these studies assessed the impact of the pandemic on dental academia and academics globally. Dental academics have an extensive network of contacts, including dental students, supporting staff, patients, and the public at large. These dentists have had extensive training on infection prevention and control, and they enjoy the respect of society because of their academic and professional backgrounds. Thus, dental academics are ideally situated to guide those around them on how to safely deal with the COVID-19 pandemic, to provide training to other dentists and dental students, and to serve as volunteer frontline staff when there is a shortage of health care personnel [13, 14].

The aim of the study was, therefore, to assess the knowledge of symptoms, modes of transmission, diagnosis, management, infection control, and dental-treatment precautions of COVID-19 disease among dental academics globally. We hypothesized that COVID-19 knowledge is higher among academics from countries with higher numbers of COVID-19 cases per million and those with extensive contact with family, students, and patients.

## Methods

This was a cross-sectional study that collected multi-country data through an online survey. The study was approved by the Research Ethics Committee of the Faculty of Dentistry, Alexandria University, Egypt (IRB 00010556)-(IORG 0008839)/6-11-2016), with further approvals from the University of Giessen in Germany (AZ: 55/20), Nigeria (IPH/OAU/12/1556), Bosnia and Herzegovina (18/4.3.26/20 and 01–952/2), Indonesia (FKGUI/IV/2020, protocol #: 090050420), Iran (IR.TUMS.DENTISTRY.REC.1399.001) and Jordan (220/132/2020). We included dental academics or educators working in dental academic institutions. Dental students (undergraduate and postgraduate) and dentists who do not work in dental educational institutions were excluded.

Table 1 lists the countries included in the study. The number of dental academics per country was estimated by using a ratio of 1:5 dental academics to dentists, based on information extracted from the World Health Organization’s (WHO) Global Health Observatory database [15]. The required number of dental academics per country to achieve statistical power was calculated, assuming 95% confidence level, 5% margin of error, and 71% adherence to infection control practices among dental academics [16].

**Table 1:** Countries included in the study, the number of recruited dental academics, and response rate, March–April 2020

Countries	Number of recruited academics	Number of responses	Response rate	N COVID-19 cases/ million
Bosnia and Herzegovina	98	58	59.2	536
Brazil	1350	118	8.7	375
Egypt	310	126	40.6	51
France	630	44	7.0	2550
Germany	1400	234	16.7	1933
India	1662	240	14.4	24
Indonesia	200	178	89	37
Iran	700	274	39.1	1127
Italy	527	62	11.8	3367
Japan	205	6	2.9	110
Jordan	100	75	75.0	44
Kenya	60	4	6.7	7
Korea	220	36	16.4	210
Libya	103	32	31.1	9
Myanmar	100	28	28.0	3
Nigeria	86	45	52.3	8
Palestine	53	27	50.9	67
Peru	150	15	10.0	1029
Saudi Arabia	90	55	61.1	654
Serbia	400	11	2.8	1031
Syria	150	17	11.3	2
Thailand	470	27	5.7	42
United Arab Emirates	77	14	18.2	1262
United Kingdom	150	63	42.0	2434
United States	6820	175	2.6	3225
Yemen	200	81	40.5	0.2
Total = 26	14,281	2045	14.3	–

We identified the countries with a large number of dentists from the Global Health Observatory [15] with the assumption that these countries also have large numbers of dental academics (there is no data available about the global distribution of dental academics). These countries were Brazil, India, the United States, China, Japan, and Germany, and we invited collaborators from these countries to join our study. To attain the greatest geographic distribution of respondents, we invited colleagues from 20 other countries to participate.

We reached the convenience sample of participants by using two strategies: First, we asked collaborators to distribute the survey to dental academics in their respective countries. Second, we scanned the official institutional websites of dental schools in the United States and Brazil where we had no collaborators and curated faculty email addresses and directly invited the addressees to take part in the survey. We aimed to include academics from as many institutions per country as possible.

The online survey tool was pilot tested with five dental academics who were excluded from the survey. They tested the content and face validity of the questionnaire and the time taken to respond to the questionnaire (average time of 4.36 min). The online survey invitation to undertake the finalized questionnaire included an introduction of the study team; the estimated time required to complete the survey; information about the right to withdraw from the survey; and details about the confidential handling of the survey information. The survey was open from March 15th to April 27th^,^ 2020.

The survey included two sections of close-ended questions. The first section had 29 questions with multiple selections allowed and assessment of six knowledge domains based on information from the WHO and the Centers for Disease Control and Prevention official websites posted during March 2020 about COVID-19 [17–19]. Six items assessed knowledge of the mode of transmission of SARS-CoV-2; 4 assessed knowledge of major warning symptoms of COVID-19; 5 assessed knowledge of treatment and management; 4 assessed items knowledge of COVID-19 diagnosis; 5 assessed knowledge of protection from COVID-19; and 5 assessed knowledge of precautions to be taken during dental treatment. The response for each item was scored either 1 (correct) or 0 (incorrect). The score for each domain was the sum of the correct responses, with domain scores ranging from zero to a maximum of 6, 4, 5, 4, 5, and 5, respectively. The total knowledge score was the sum of the scores for all domains, with possible scores ranging from zero to 29.

The second section had 12 questions that generated information on respondents’ background: age; sex; living arrangements; country of practice; specialization; highest academic degree obtained; number of years in academia; number of courses taught/coordinated; average number of students per semester; average number of patients attended to in the clinic per day; training on the handling of public health emergencies; and administrative role. Appendix 1 is the questionnaire used for this study.

Using Survey Monkey®, an online survey platform, we prepared the links to the survey with settings to ensure that it would be anonymous, that participants could change their answers freely before they choose to submit, and that it was not time-limited. One submission per electronic device was allowed. We created the questionnaire in English and translated it when needed to the language of the dental academia in specific countries, we had Portuguese and Farsi translations. The translating was done by native dentists with back translation to English to ensure accuracy. Links were sent to eligible participants through email or social media groups of academics only, and no incentives or rewards were offered. The first invitation to participate was distributed from March 15th to 27th 2020, and follow-up reminders were sent out from April 8th to 14th [20].

We calculated the percentages of correct responses and plotted them as bar graphs. We assessed the internal consistency with Kuder-Richardson formula 20 (K-R 20), a modification of Cronbach’s alpha [21]. We compared the knowledge domain percentage scores using multivariate analysis of variance (MANOVA), controlling for country effect, to assess whether there were differences in the knowledge about various aspects of COVID-19. We used the linear mixed-model procedure in SPSS version 23.0 to construct unadjusted multilevel linear regression models, in which we entered the explanatory variables one at a time. Multilevel linear regression models were appropriate to use to account for the clustering of academics within countries: countries’ differences were expected to be associated with the level of knowledge. The outcome variable was the total knowledge score. The explanatory variables were at the individual level (background information) and country-level (the number of COVID-19 cases per million population obtained from Worldometer website (Table 1) [2].

We developed an unconditional model, including no explanatory variables, to calculate the baseline variance due to random differences among countries. In the second step, we entered individual and country-level variables that were significantly associated with the outcome variable in the unadjusted models into a multilevel model as fixed effects and used country as a random effect. We calculated regression coefficients (B), 95% confidence intervals (CIs), residual variance, deviance (as − 2 log-likelihood (LL)), X^2^ test to assess improvement in the goodness of fit relative to the unconditional model and increase in pseudo R^2^ [22]. Statistical significance was set at 5%.

## Results

There were 2045 responses from 26 countries. The response rate ranged from 2.6% in the United States to 89% in Indonesia, with an overall response rate of 14.3% (Table 1). Table 2 shows that 1099 (54.7%) participants were female; 706 (34.5%) were 25–35 years old; 1301 (63.6%) lived with partner/spouse; 1735 (84.8%) were specialists; 897 (43.9%) were PhD holders; 597 (29.2%) have been in academia for 5–10 years; 2.3 courses were coordinated/taught on average; 663 (32.4%) had 50–100 students per semester; 943 (46.1%) managed 1–10 patients per day; 1064 (52%) had no previous training in public health emergencies; and 1073 (52.2%) had administrative positions. The average number of COVID-19 cases per million population in the participating countries was 972.9, with a median of 375 COVID-19 cases per million.

**Table 2:** Individual and country-level factors of participating dental academics and their association with knowledge score

Factors		N (%)	Knowledge score EM (SE)	B (95% CI)	*P* value
Individual-level factor					
Sex	Male	911 (45.3)	73.01 (0.37)	−0.47 (−1.45, 0.51)	0.34
	Female	1099 (54.7)	73.48 (0.34)	Reference	–
Age	25–35	706 (34.5)	72.85 (0.42)	−2.85 (−5.36, − 0.34)	0.03*
	> 35–45	664 (32.5)	73.49 (0.43)	−2.21 (−4.72, 0.31)	0.09
	> 45–55	354 (17.3)	72.87 (0.59)	−2.83 (−5.47, − 0.19)	0.04*
	> 55–65	236 (11.5)	73.29 (0.73)	−2.41 (−5.17, 0.36)	0.09
	> 65	85 (4.2)	75.70 (1.21)	Reference	–
Living arrangements	With parents	335 (16.4)	70.93 (0.61)	−1.99 (−3.84, −0.13)	0.04*
	With partner/ spouse	1301 (63.6)	74.17 (0.31)	1.25 (−0.30, 2.79)	0.11
	Shared accommodation	76 (3.7)	72.01 (1.27)	−0.91 (−3.78, 1.96)	0.53
	Other	101 (4.9)	70.50 (1.19)	−2.42 (−5.01, 0.17)	0.07
	Alone	232 (11.3)	72.92 (0.73)	Reference	–
Specialization	Specialist	1735 (84.8)	21.33 (0.08)	0.58 (0.20)	0.003*
	No-specialist	310 (15.2)	20.74 (0.18)	Reference	–
Highest academic degree	PhD	897 (43.9)	73.95 (0.37)	1.88 (0.62, 3.15)	0.004*
	MSc	703 (34.4)	73.06 (0.42)	1.00 (−0.33, 2.32)	0.14
	BDS	445 (21.8)	72.07 (0.53)	Reference	–
Number of years in academia	< 5	530 (25.9)	73.25 (0.48)	−0.16 (−1.62, 1.30)	0.83
	5–10	597 (29.2)	72.93 (0.46)	−0.48 (− 1.91, 0.94)	0.51
	11–20	527 (25.8)	73.43 (0.49)	0.02 (−1.44, 1.48)	0.98
	21+	391 (19.1)	73.41 (0.56)	Reference	–
Number of courses coordinated: mean (SD)		2.3 (1.7)	–	−0.21 (−0.48, 0.07)	0.15
Number of students per semester	None	131 (6.4)	70.20 (0.97)	Reference	–
	1–49	506 (24.7)	73.96 (0.49)	1.09 (0.47, 1.71)*	0.001*
	50–100	663 (32.4)	73.17 (0.43)	0.86 (0.25, 1.47)*	0.005*
	101–200	370 (18.1)	73.30 (0.58)	0.90 (0.25, 1.54)*	0.006*
	201+	375 (18.3)	73.36 (0.57)	0.92 (0.27, 1.56)*	0.005*
Number of patients seen daily	None	224 (11.0)	72.17 (0.74)	Reference	–
	1–10	943 (46.1)	72.51 (0.36)	0.10 (−0.37, 0.57)	0.68
	11–20	525 (25.7)	74.78 (0.48)	0.76 (0.25, 1.26)*	0.003*
	21–30	156 (7.6)	72.61 (0.89)	0.13 (−0.53, 0.79)	0.70
	31+	197 (9.6)	74.29 (0.79)	0.61 (−0.003, 1.23)	0.05
Training for public health emergencies	No	1064 (52)	73.43 (0.34)	0.41 (−0.56, 1.38)	0.40
	Yes	981 (48)	73.02 (0.36)	Reference	–
Administrative position	No	972 (47.5)	73.34 (0.36)	0.20 (−0.77, 1.17)	0.68
	Yes	1073 (52.5)	73.14 (0.34)	Reference	–
Country-level factor					
Number of cases COVID-19 per million population: mean (SD)		972.9 (1123.1)	–	0.002 (0.002, 0.003)	< 0.0001*

Abbreviations: *EM* Estimated means based on unadjusted multilevel linear models with country as a random effect, *SE* Standard error, *B* Regression coefficient, *CI* Confidence interval

*: Statistically significant at *P* < 0.05

**Fig. 1:**
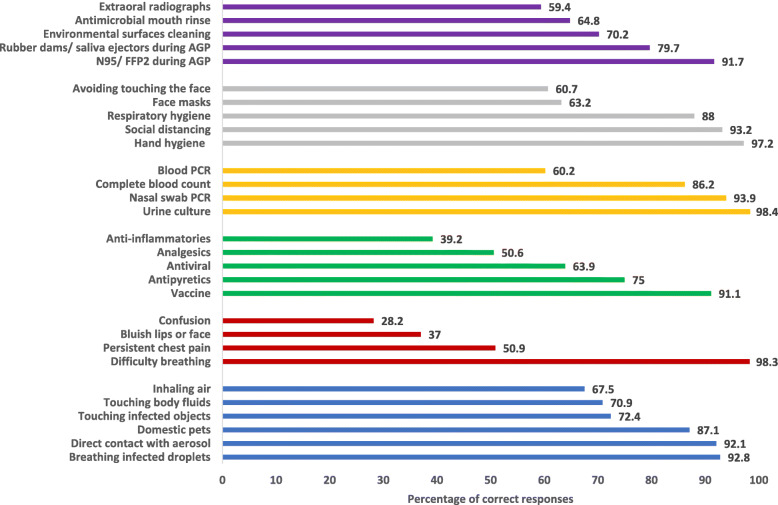
Percentage of correct responses for items of transmission (blue), symptoms (red), treatment (green), diagnosis (orange), protection (grey), and dental precautions (purple)

Figure 1 illustrates the levels of dentists’ COVID-19 knowledge. About 92% knew that COVID-19 could be transmitted through breathing infected droplets and direct contact with aerosols. Almost all participants (98.3%) identified difficulty in breathing as a warning symptom, while only 28.2% identified confusion as a warning symptom. About 91% knew that there is no COVID-19 vaccine, and 63.9% knew there is till the present time no COVID-19 antiviral therapy. Almost all participants (98.4%) ruled out urine culture as a method to diagnose COVID-19. Also, 97.2% of respondents identified hand hygiene and 60.7% identified avoiding touching the face as methods of protection against infection. Most participants (91.7%) identified the use of N95/FFP2 masks during aerosol-generating procedures, and 59.4% identified the use of extra-oral rather than intraoral radiographs as protective measures when treating patients suspected to have COVID-19 infection.

**Fig. 2:**
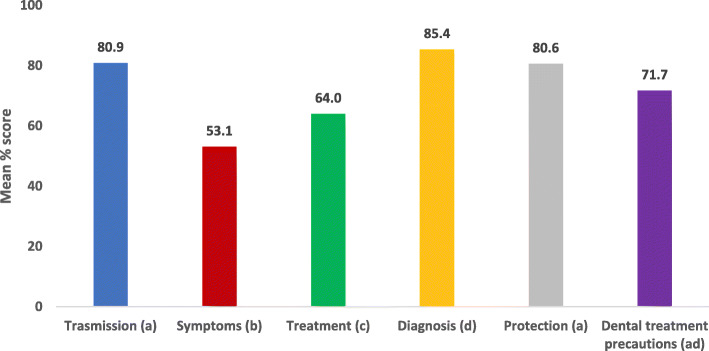
Percent scores of transmission, warning symptoms, treatment, diagnosis, protection, and dental treatment precautions. Letters a, b, c, and d below the x-axis next to the domain label denote statistically significant differences. All scores were adjusted for the country

Figure 2 illustrates the mean percentage scores in each of the 6 knowledge domains. The percent scores of knowing disease symptoms (mean = 53.1%) and treatment modalities (mean = 64%) were significantly different from each other (*P* <  0.0001) and significantly lower than all other scores (*P* <  0.0001). The percent scores of transmission (mean = 80.9%), protection (mean = 80.6%), and dental treatment precautions (71.7%) domains were not significantly different from each other (*P* = 0.99). The percent score of diagnosis (mean = 85.4%) and dental treatment precautions (mean = 71.7%) were not significantly different from each other. The percent score of knowing COVID-19 diagnosis methods was significantly higher than the scores of knowledge about transmission, symptoms, treatment, and protection methods (*P* <  0.0001).

K-R 20 for all items was 0.58. The mean (SD) percent knowledge score was 73.2 (11.2) %. Table 2 shows that in the unadjusted multilevel models, the percent knowledge score was significantly associated with age, living arrangements, specialization, highest academic degree obtained, number of students per semester, number of patients seen per day, and number of COVID-19 cases per million population (*P* < 0.05). Participants who were 25–35 years old (*P* = 0.03) and 45–55 years old (*P* = 0.04) had significantly lower scores than did participants who were > 65 years old. Those living with their parents had significantly lower scores than did those living alone (*P* = 0.04). Specialists had significantly higher scores than did non-specialists (*P* = 0.003). PhD holders had significantly higher scores than did those with only Bachelor of Dental Science (BDS) degree (*P* = 0.004). Those who taught students had significantly higher scores than did those who did not teach students (*P* < 0.05). Those who saw 11–20 patients daily had higher knowledge than did those who saw no patients (*P* = 0.003). Participants from countries with a higher number of COVID-19 affected people per million population had significantly higher scores than did those with a lower number of affected people (*P* < 0.0001).

Table 3 shows that the full model including individual- and country-level factors showed significant improvement in fit over the unconditional model (P of X^2^ < 0.0001), with an increase in pseudo R^2^ of 35.5%. The full model had lower deviance (10,488.18) than did the unconditional model). Also, those living with a partner/spouse had significantly higher knowledge scores than did those living alone (B = 0.48, 95% CI = 0.03, 0.92). PhD degree holders had significantly higher scores than did academics with only BDS (B = 0.48, 95% CI = 0.07, 0.89). Participants who had 21–30 patients per day had significantly higher scores than did those seeing no patients (B = 0.65, 95% CI = 0.15, 1.16). Participants from countries with a higher number of COVID-19 cases per million population had significantly higher scores than did those from countries with a lower number of COVID-19 cases (B = 0.0007, 95% CI = 0.0005, 0.0008).

**Table 3:** Multilevel models for individual and country-level factors affecting dental academics knowledge of COVID-19

Factors		Unconditional model^a^	Full model^b^ B (95% CI)
Individual factors			
Age	25–35 versus > 65	–	0.28 (−0.47, 1.02)
	> 35–45 versus > 65		0.15 (−0.60, 0.90)
	> 45–55 versus > 65		−0.21 (− 0.98, 0.56)
	> 55–65 versus > 65		− 0.39 (−1.18, 0.41)
Living arrange	With parents versus alone		0.02 (− 0.52, 0.57)
	With partner/ spouse versus alone		0.48 (0.03, 0.92)*
	Shared accommodation versus alone		0.12 (−0.69, 0.94)
	Other verses alone		−0.25 (− 0.99, 0.49)
Specialty	Specialist versus non-specialist		0.34 (−0.06, 0.75)
Highest degree obtained	PhD versus BDS		0.48 (0.07, 0.89)*
	MSc versus BDS		0.29 (−0.12, 0.70)
Number of students per semester	1–49 versus none		0.52 (−0.14, 1.18)
	50–100 versus none		0.38 (−0.27, 1.04)
	101–200 versus none		0.16 (−0.46, 0.78)
	201+ versus none		0.50 (−0.13, 1.12)
Number of patients seen daily	1–10 versus none		0.55 (−0.06, 1.17)
	11–20 versus none		−0.04 (− 0.69, 0.61)
	21–30 versus none		0.65 (0.15, 1.16)*
	31+ versus none		0.22 (−0.25, 0.68)
Country factor			
COVID-19 cases per million			0.0007 (0.0005, 0.0008)*
Residual		15.17	9.78
Deviance		15,670.16	10,488.18
P of X^2^ of improved fit		–	< 0.0001*
Increase in R^2^		–	35.5%

a: Unconditional model: no explanatory factors included- country included as a random effect

b: Full model: individual and country factors included with mutual adjustment- country included as a random effect

Abbreviations: *B* Adjusted regression estimates, *CI* Confidence interval

## Discussion

The study found that dental academics’ knowledge of the mode of transmission, methods of diagnosis, and preventive dental practices for COVID-19 was better than the knowledge of the symptoms of COVID-19 and its treatment. Factors associated with better knowledge included having more human contacts (living with spouse/partner and higher patient load), having a PhD degree, and the magnitude of the country’s COVID-19 pandemic. Thus, the study hypothesis was partly substantiated.

A major strength of the study is the large sample size and diversity of respondents’ countries, which allowed us to demonstrate between-country differences in dental academics’ knowledge of the pandemic based on the size of the country pandemic. The rapidly evolving nature of the pandemic means that information assessed in this study may have already become outdated by the time we conducted the analysis. The validity of our conclusions is, therefore, time relevant. However, this analysis focuses on dental academics and provides useful insights. The findings are valuable for designing and planning continuing education programs for dental academics on COVID-19 and for identifying areas where emphasis on updated information is needed.

In the present study, the internal consistency of the items used to measure knowledge just reached the acceptable level and was rather low. This may be explained by the inclusion of measures of various domains of knowledge that were not well understood by participants at the time of conducting this study. The study findings can help develop COVID-19-related training curricula when pre-training evaluation cannot be conducted. Most studies that had assessed the knowledge of dental personnel focused on dentists [9–12, 23, 24], with little information about dental academics. Therefore, direct comparison with multi-country studies about academics is not possible. Multi-country studies [11, 25] reported scores of dentists’ knowledge of COVID-19 that were lower than those reported for dental academics in the current study. Dental academics in this study also recorded higher scores on knowledge of COVID-19 than did dentists in Saudi Arabia, who reported knowledge of symptoms and diagnosis of MERS-CoV [24], knowledge of MERS-CoV treatment, and awareness about the availability of a vaccine for MERS-CoV [23]. The higher knowledge about COVID-19 compared with MERS-CoV in the present study may be attributed to the global nature of the COVID-19 pandemic compared to the one-region MERS-CoV outbreak, which made it possible for dental academic to receive updated information through webinars, social media, education channels, and from the extensive media coverage of the race among countries and big pharmaceutical companies to develop a COVID-19 vaccine [26]. Academics are also more apt to use the information for research purposes and are therefore more likely to be receptive to information than are non-academic dentists. This difference should be explored further.

The rapid pace at which the COVID-19 outbreak and information about it changes emphasizes the importance of credible sources of information [27]. International agencies with global reach, such as the WHO, are offering open online courses on COVID-19 in multiple languages to ensure that professionals, such as dental academics, keep up to date with new information [28]. The availability of information is especially important in regions where the shortage of health care professionals calls for training dentists/ dental academics as first responders in public health emergencies [29].

In this study, dental academics who had a greater risk of exposure to SARS-CoV-2 -- through higher patient load and a larger social network – had better knowledge of COVID-19. A higher perception of risk may lead academics to seek COVID-19 information, which may explain why dental academics in countries with a higher number of COVID-19 cases per million population had better knowledge of COVID-19; likely, there was motivation to know more since their perception of the risk of contracting infection was higher. Their better knowledge may also be attributed to greater exposure to media information about COVID-19 [30, 31]. The relationship between COVID-19 risk perception and knowledge should be explored in future studies.

This study also revealed that PhD holders had higher knowledge scores than did academics with BDS degrees only, as had been found in a prior study with dentists [11]. This finding may be related to the comprehensive insight of PhD holders about disease processes and management [32] in addition to their research orientation and interest in reading more journal articles.

This study has limitations: The first is the cross-sectional design, which cannot cover the change in dental academics’ knowledge at points in time during the pandemic: with more exposure to news of the pandemic, their knowledge may change. Thus, our results may underestimate knowledge over time. In addition, the convenience sampling may reduce statistical representativeness. However, convenience sampling strategy is the only feasible method to sample academics in the absence of a framework listing dental schools and academics worldwide. The low response rate from some countries limits the generalizability of the study findings to those low-response countries. Low response may be explained by the academics being busy with online teaching [33], and the saturation occurring due to exposure to multiple surveys about COVID-19. We addressed this low response rate by sending reminders, using personalized emails, and communicating with academics directly through in-country collaborators. Previous studies also reported low response in surveys conducted among health professionals and for online surveys in general, and our study response rate falls with the rate reported for online surveys [34]. Despite these limitations, the inclusion of a diverse group of dental academics from countries with various income levels, geographic locations, and educational systems increases the generalizability of the findings.

## Conclusion

Dental academics from several countries around the world had good knowledge of COVID-19 though they were less informed about COVID-19 symptoms than they were of its diagnostic and dental treatment precautionary methods. Academics with greater risks of contracting COVID-19 and those with extensive social networks, which may increase their risk of exposure to infection, also had better knowledge of COVID-19. The differences in the knowledge of COVID-19 domains can inform the development of training curricula for dental academics. This knowledge would improve multi-disciplinary and inter-sectoral collaborations between academics from different countries to help address the global pandemic.

## Data Availability

The dataset used in this research is available at synapse.org, under the title: Knowledge of Dental Academics about the COVID-19 Pandemic. Synapse ID: syn22295368. Username: @NourAmmar.

## Appendix

Knowledge of dental academics about COVID-19 infection questionnaire.

This questionnaire is targeting academics in dental institutions. Please respond to it only if you are currently working in a dental academic institution. We are assessing the knowledge of dental academics regarding the COVID-19. Your responses are confidential and cannot be traced to you. They will not be shared with anyone, and only the research team will have access to them.

(*indicates a correct response)

1. Which of the following can transmit COVID-19 virus to compromised skin or mucous membranes? (select all that applies)

- Inhaling air from an infected patient*
- Touching body fluids of an infected patient*
- Direct contact with aerosol splattered during a dental procedure in an infected patient*
- Domestic pets
- Touching objects that have been touched by an infected person*
- Breathing in droplets exhaled or coughed from an infected person*

2- Which of the following are major emergency warning symptoms in patients with COVID-19 infection that require immediate medical attention? (select all that applies)

- Persistent pain or pressure in the chest*
- Difficulty breathing or shortness of breath*
- Bluish lips or face*
- New onset of confusion or inability to arouse*

3- Which of the following is used to treat COVID-19 infection? (select all that applies)

- Antipyretics*
- Anti-inflammatories*
- Analgesics*
- Vaccine
- A new antiviral for COVID-19

4- Which of the following methods is used to diagnose COVID-19? (select all that applies)

- Real-time PCR of blood samples
- Culture of urine sample
- Complete blood count (CBC)
- Real-time PCR of throat/ nasal swabs*

5- Which of these are the 3 best methods to protect against infection? (Please, select 3)

- Hand hygiene*
- Social distancing*
- Practicing respiratory hygiene
- Avoiding touching the face*
- Face masks

6- According to the WHO guidelines, in case of patients infected with/ suspected of COVID-19 infection, which of the following should be done during dental treatment? (select all that applies)

- During aerosol-generating procedures (AGP), Respirator N95, FFP2 standard, or equivalent use is recommended*
- During AGP, rubber dams and saliva ejectors should be used*
- Preoperative antimicrobial mouth rinse is recommended*
- Extraoral radiographs are recommended over intraoral radiographs*
- The recommended agent for environmental surfaces cleaning is soap and water.

7- Gender

- Male
- Female
- No reponse

8- Age

- 25–35
- 36–45
- 46–55
- 56–65
- 66+

9- Home/living arrangements

- Live alone
- Live with parents
- Live with partner/spouse
- Live in shared accommodation
- Other

10- Country
11- Specialty
12- Highest academic degree

- BDS (or equivalent)
- M.Sc (or equivalent)
- PhD (or equivalent)

13- Number of years in academia

- < 5
- 5–10
- 11–20
- 21+

14- Number of courses you coordinate

- None
- 1
- 2
- 3
- 4
- 5 or more

15- Number of students you deal with per semester

- None
- 1–49
- 50–100
- 101–200
- 201+

16- Number of patients you deal with per day

- None
- 1–9
- 10–20
- 21–30
- 31+

17- Did the faculty staff receive any training on dealing with public health emergencies?

- Yes
- No

18- Do you have any administrative roles?

- Yes
- No

## Abbreviations

WHO: World health organization
COVID-19: The 2019 novel coronavirus
SARS-CoV 2: Severe acute respiratory syndrome coronavirus 2
CDC: Centers for disease control and prevention
MANOVA: Multivariate analysis of variance
